# Long-Term Persistence of *Yersinia pseudotuberculosis* in Entomopathogenic Nematodes

**DOI:** 10.1371/journal.pone.0116818

**Published:** 2015-01-30

**Authors:** Samuel Gengler, Anne Laudisoit, Henri Batoko, Pierre Wattiau

**Affiliations:** 1 Veterinary and Agrochemical Research Centre (VAR), Brussels, Belgium; 2 Institute of life sciences, Catholic University of Louvain-la-Neuve (UCL), Louvain-la-Neuve, Belgium; 3 Antwerp University, Evolutionary Biology, 171, Groenenborgerlaan, 2020 Antwerpen, Belgium; 4 Institute of Integrative Biology, School of Biological Sciences, University of Liverpool, Liverpool, United Kingdom; University of Helsinki, FINLAND

## Abstract

Entomopathogenic nematodes (EPNs) are small worms whose ecological behaviour consists to invade, kill insects and feed on their cadavers thanks to a species-specific symbiotic bacterium belonging to any of the genera *Xenorhabdus* or *Photorhabdus* hosted in the gastro-intestinal tract of EPNs. The symbiont provides a number of biological functions that are essential for its EPN host including the production of entomotoxins, of enzymes able to degrade the insect constitutive macromolecules and of antimicrobial compounds able to prevent the growth of competitors in the insect cadaver. The question addressed in this study was to investigate whether a mammalian pathogen taxonomically related to *Xenorhabdus* was able to substitute for or “hijack” the symbiotic relationship associating *Xenorhabdus* and *Steinernema* EPNs. To deal with this question, a laboratory experimental model was developed consisting in *Galleria mellonella* insect larvae, *Steinernema* EPNs with or without their natural *Xenorhabdus* symbiont and *Yersinia pseudotuberculosis* brought artificially either in the gut of EPNs or in the haemocoel of the insect larva prior to infection. The developed model demonstrated the capacity of EPNs to act as an efficient reservoir ensuring exponential multiplication, maintenance and dissemination of *Y. pseudotuberculosis*.

## Introduction

Entomopathogenic nematodes (EPNs) are microscopic soil worms exclusively feeding on insect preys. They have the ability to cause death in a huge variety of insects, making them powerful candidate biopesticides in agriculture and horticulture [1,2]. EPNs owe their insecticidal properties to symbiotic bacteria belonging to two genera of *Enterobacteriaceae*, namely *Xenorhabdus* and *Photorhabdus*. These bacteria are hosted in the gastro-intestinal tract of the nematode–located in an intestinal vesicle in the case of *Xenorhabdus* [3] – at the infectious free-living stage, called infective juveniles (IJs). Upon invasion of an insect prey, the symbiotic bacteria are expelled from the IJ’s digestive tract. These bacteria multiply in the insect haemocoel and release insecticidal toxins as well as degradative enzymes able to digest the insect macromolecules, thereby feeding their EPN partners which mature to the adult stage through 4 larval stages named J1 to J4 and undergo several reproduction cycles [4]. Moreover, the symbiont prevents microbial competitors growth inside the insect’s cadaver by releasing antibiotic and antifungal compounds [5]. After all insect macromolecules have been exhausted, a few symbiotic bacteria enter the intestinal tract of the mature IJs just before they emerge from the dead larva and seek another prey [6,7].

While a few EPNs are generally sufficient to kill an insect prey, up to half a million of IJs can emerge from a single infected host upon completion of their reproductive life cycle inside the insect cadaver [8]. Each of these freshly emerged IJs is able to infect a new insect prey. IJs can survive in the soil for several months thanks to their protective cuticle and a huge lipid supply they can store [9].

In 2008 Heermann and Fuchs have shown that *Photorhabdus luminescens*, the bacterial symbiont of *Heterorhabditis bacteriophora*, shares a number of unique genes with the taxonomically related, yet ecologically different, *Yersinia enterocolitica* [10]. The shared genes are for some of them clustered in so-called “High Pathogenicity Islands” described in *Enterobacteriaceae* including *Yersinia* [11]. Many of these genes are either involved in pathogenicity toward insects, like the insecticidal toxin complex (Tc) [12], or in colonisation of eukaryotic cells, like the YplA phospholipase [13]. The recently discovered type 6 secretion system (T6SS) involved in toxin secretion and in mutualism between bacteria [14] is also conserved between *P. luminescens* and *Y. enterocolitica* [10]. Unlike *P. luminescens* which can cause casual infection in humans [15], *Y. enterocolitica* as well as *Y. pseudotuberculosis* are mammalian pathogens causing gastro-intestinal diseases in infected hosts. These two *Yersiniae* are regularly isolated from meat–especially pork meat–and root vegetables [16,17]. However, they have also been found in the gut lumen of adult flies and fly larvae, suggesting that they can use insects as passive vectors [18,19,20]. In addition, in vitro experiments have shown that both *Y. enterocolitica* and *Y. pseudotuberculosis* are able to colonize insect cells [21] and even to kill insect larvae like *Galleria mellonella* [22]. It is well known that *Yersinia pestis*, the third mammalian pathogenic *Yersinia* and etiological agent of plague, is able to colonize insects since it uses fleas as vectors. Hinnebusch et al. demonstrated the essential implication of the *Yersinia* murine toxin (Ymt) in flea colonisation [23]. Interestingly, it has been suggested that *Y. pestis* acquired *ymt* gene from *P. luminescens* or from a close relative [24]. Moreover, *Photorhabdus asymbiotica* which can infect either insects or humans, possesses a plasmid related to pMT-1 found in *Y. pestis* [25].

Besides their animal hosts, *Y. enterocolitica* as well as *Y. pseudotuberculosis* are commonly found in water, soil and vegetables [16,17,26]. Several studies have shown that *Y. pestis* can also be found in soil [27,28]. Moreover, several experiments have highlighted the survival of *Y. enterocolitica, Y. pseudotuberculosis* and *Y. pestis* in free living soil amoeba [29,30]. Since pathogenic *Yersiniae* are able to persist in soil and are phylogenetically very close to the bacterial symbionts of EPNs, we wondered whether *Yersiniae* would be able to intrude the symbiotic relationship associating EPNs and their natural symbiont. In order to test this hypothesis, we used an experimental model consisting of insect larvae of the species *Galleria mellonella* used as prey for an African species of entomopathogenic *Steinernema* hosting its natural *Xenorhabdus* symbiont as well as a *Y. pseudotuberculosis* field isolate naturally resistant to the anti-microbial compounds produced by *Xenorhabdus*. We show that *Y. pseudotuberculosis* can be successfully transmitted by the EPN carrier inside an insect larva in which it persists and multiplies. Moreover, EPNs emerging from the insect cadaver after 10 to 15 days where found to host large numbers of *Y. pseudotuberculosis* cells in their gastro-intestinal tract. These EPNs were in turn able to transmit *Y. pseudotuberculosis* to a new insect larva and so on for at least 7 successive infectious cycles (14 weeks). If they turn out to have an ecological significance, these findings may reveal an unexpected biotic reservoir for the long-term persistence and dissemination of pathogenic Yersiniae in the environment.

## Material and Methods

### Bacterial strains, plasmids and growth conditions

*Enterobacteriaceae* were grown in LB liquid broth with strong agitation (150rpm) or on LB agar or McConkey agar plates. The NBTA plates (nutrient agar supplemented with 25 mg l^−1^ bromothymol blue and 40 mg l^−1^ triphenyltetrazolium chloride) [31] were also used to check the phase I of the *Xenorhabdus* species used in this study. The incubation temperature was 37°C except for *Yersiniae* and *Xenorhabdus* sp. which were grown at 28°C. Antibiotics were added at the following concentrations: Kanamycin (Km): 30μg ml^−1^, Nalidixic acid (Nal): 25μg ml^−1^, Streptomycin (Sm): 50μg ml^−1^; Ampicillin (Ap): 100μg ml^−1^. Nalidixic-acid resistant (Nal^R^) bacteria were obtained in three consecutive steps by plating 10^7^ to 10^9^ CFU per agar plate supplemented with increasing concentrations of Nalidixic acid (5μg/ml; 20μg/ml; 50μg/ml). Bacterial strains used in this study are listed in Table 1.

**Table 1:** List of bacterial strains used in this study.

**Strains**	**Origin (Reference)**	**Description**
*Escherichia coli* S17-1λPir	NCCB * (Simon, R. et al., Biotechnol. (1983) 1: 784–791, McFarlane, G.J.B. et al, J. Microbiol. Methods (1987) 6: 301–305)[51,52]	λ lysogenic S17-1 derivative expressing the π protein required for replication of plasmids carrying *oriR6K*; Sm^R^
*E. coli* 17WP	This work (de Lorenzo, V et al., J. Bacteriol. (1990) 172(11):6568–72)[53]	*E. coli* S17-1 λPir carrying pUT-miniTn5-*gfpmut2*, a transposon delivery suicide vector. GFP mini-transposon delivery strain; Sm^R^/Ap^R^/Km^R^
*E. coli* SM10 λPir	(Miller & Mekalanos, J. Bacteriol. (1988) 170(6):2575–83)[34]	λ lysogenic *E. coli* derivative expressing the π protein required for replication of plasmids carrying *oriR6K*; Km^R^
*E. coli* 10WP	This work	*E. coli* SM10 λPir carrying *mCherry* flanked by *gfp-mut2* moieties and cloned into pKNG101. Fluorescence cassette replacement vector termed pSGCG; Km^R^, Sm^R^, *sacBR* ^+^
*E. coli* strain EC26-KH-2010	VAR^§^	Vero-toxigenic *Escherichia coli* of the O157 serogroup isolated from a Belgian calf in 2010
*E. coli* strain VT02	This work	Nalidixic-acid resistant mutant of EC26-KH-2010
*E. coli* strain VT03	This work	VT02 carrying a randomly inserted Gfpmut2-transposon; Nal^R^/Km^R^
*Xenorhabdus* sp. strain TZ01	Anne Laudisoit, This work (Mwaitulo et al., Int. J. Trop. Insect Sci. (2011) 26(4):214–226)[37]	Symbiotic bacterium retrieved from *Steinernema tanzaniensis* nematodes isolated from Tanzanian soil
*X*. sp. strain TZ02	This work	Nalidixic-acid resistant mutant of TZ01
*X*. sp. strain TZ03	This work	TZ02 carrying a randomly inserted Gfpmut2-transposon; Nal^R^/Km^R^
*Yersinia pseudotuberculosis* strain IP2777	Institut Pasteur Lille (Derbise et al., J. Infect. Dis. (2013) 207(10):1535–43)[54]	Human clinical isolate, serotype O1
*Y. pseudotuberculosis* strain 2008/04429	VAR^§^	Isolated from a rabbit cadaver (Belgium)
*Y. pseudotuberculosis* strain 4N1	This work	Nalidixic acid-resistant mutant of 2008/04429
*Y. pseudotuberculosis* strain 4N1G	This work	2008/00429 4N1 carrying a Gfpmut2-transposon inserted in the fimbrial A protein A gene (see M&M); Nal^R^/Km^R^
*Y. pseudotuberculosis* strain 4N1C	This work	4N1G with *mcherry* replacing *gfpmut2* in the mini-transposon following allelic exchange using pSGCG.
*Y. enterocolitica* strain VAR08/02	VAR^§^	Pig Isolate belonging to serotype O3
*Y. enterocolitica* strain YE02	This work	Nalidixic-acid resistant mutant of VAR08/02
*Y. enterocolitica* strain YE03	This work	YE02 carrying a randomly inserted Gfpmut2-transposon; Nal^R^/Km^R^
*Salmonella enterica* subsp. *enterica* sv. Enteritidis strain 2011/03561	VAR^§^	Field isolate of poultry origin (Belgium)
*S*.Enteritidis strain SE02	This work	Nalidixic-acid resistant mutant of 2011/03561
*S*.Enteritidis strain SE03	This work	SE02 carrying a randomly inserted Gfpmut2-transposon; Nal^R^/Km^R^
*Serratia marcescens* strain EE016	This work	Isolated from a *Steinernema* sp. MW8B-infected *G. melonella* larva
*Ochrobactrum tritici* strain EE10.1	This work	Isolated from a *Steinernema* sp. MW8B-infected *G. melonella* larva

* NCCB, The Netherlands Culture Collection of Bacteria, Utrecht, The Netherlands.

^§^ VAR, Veterinary and Agrochemical Research Center, Brussels, BELGIUM.

Some *Enterobacteria*, listed in table 1, were fluorescently labelled with GFP-mut2 [32] using a mini-Tn*5* transposon [33]. Mini-transposon labelling was conducted by conjugating a nalidixic-acid resistant variant of the target bacterium with *E. coli* S17/1 λ pir hosting a transposon delivery suicide vector [34]. Transconjugants were isolated on selective agar plates and tested for GFP fluorescence. Integration of *gfp-mut2* was further confirmed by PCR with primers mut2-GFP_F (GGG ATC TTT CGA AAG GGC AGA TTG TGT GG) and mut2-GFP_R (GGA GAG GGT GAA GGT GAT GCA ACA TAC GG). The size of the amplified fragment was 543 bp. For dual labelling experiments, the *gfp-mut2* gene of *Y. pseudotuberculosis* 4N1G was substituted for *mCherry*, encoding a red-fluorescent protein, by allelic exchange. The replacement cassette consisted in *mCherry* flanked by the beginning and the end of the *gfp-mut2* nucleotide sequence. The upstream and downstream flanking parts consisted of 244 and 223 base pairs of *gfp-mut2*, respectively, obtained by PCR amplification. A ribosome binding site was added upstream of the *mCherry* open reading frame to ensure optimal translation. The replacement cassette was cloned into the mobilizable suicide vector pKNG101, which confers resistance to streptomycin and carries the counter-selectable marker *sacBR* [35]. The recombinant suicide plasmid termed pSGCG was then transferred to *Y. pseudotuberculosis* 4N1G from the conjugative strain *E. coli* SM10λpir. Allelic exchange was conducted in two steps. Initial integration of pSGCG was first selected on specific agar plates containing streptomycin (100μg ml^−1^). After purification of the recombinant strain, allelic exchange was selected on agar plates containing sucrose (100μg ml^−1^) and recombinant colonies expressing mCherry but not GFP were controlled by both epifluorescence microscopy and PCR.

### Mini-Tn*5* transposon insertion mapping

The mini-Tn*5* transposon used here to tag the *Yersinia pseudotuberculosis* derivatives 4N1G and 4N1C was mapped by TAIL-PCR using the method of Liu and Wittier [36] and by sequencing the amplified fragment. The mini-Tn*5*-gfp was found inserted in the chromosome at codon 60 of the fimbrial A protein gene in the same transcriptional orientation (ORF YPK_0694 as described in the annotated genome of *Y. pseudotuberculosis* YPIII, Accession number NC_010465.1). The transposon-specific primers used for TAIL-PCR were the following: SP1: CGC GAA AGT AGT GAC AAG TGT TGG CCA TGG; SP2: GTA TAA CAT GTC TTA TAC GCC CGT GTC AAC C; SP3: AGA TCC CCG GGT ACC GAG CTC GAA TTC GCG. The arbitrary degenerated primers used here were the same described by Liu and Whittier [36]. Final confirmation of the insertion point of the mini-Tn*5* transposon was obtained by amplifying chromosomal fragments covering part of the transposon and part of the fimbrial A protein gene using the PCR primers SP1, SP2 or SP3 together with the fimbrial-specific primer CCG GTT CTA TCA TTG AAG CAC CTT GTT C.

### Growth and maintenance of nematodes

*Steinernema* sp. MW8B isolated from Tanzanian soil [37] was used as model nematode allover the experiments. *Steinernema* sp. MW8B is symbiotically associated with *Xenorhabdus* sp. strain TZ01. Nucleotide sequence of the TZ01 16S ribosomal RNA gene (GenBank accession JQ687358.1) is equally similar, though not 100% identical, to that of *X. ehlersii, X. budapestensis, X. griffiniae* and *X. kozodoii*. Nematode stocks (Infective juvenile stage) were maintained by successive passages through the last larval stage of the greater wax moth, *Galleria mellonella*. Infection of the larvae was conducted by incubating 500 to 1000 *Steinernema* sp. MW8B IJs suspended in 1ml physiological water (NaCl 9g L^−1^) with four to six larvae confined in a closed Petri dish. Upon emergence from the dead larvae which occurred after 10 ± 2 days later, IJs were collected and stored at room temperature in physiological water.

### *Galleria mellonella* in vitro infection model

For the first infection cycle, 6 *G. mellonella* larvae were injected with 10^6^ CFU of the studied bacterium (*Yersinia* sp., GFP-labelled or not) using sterile 1-ml syringes bearing 0.3 × 13mm needles (Becton Dickinson). Injection was performed on the side of the larvae at the basis of the 8^th^ segment. After incubation with ±750 *Steinernema* sp. MW8B IJs (125 IJs/larva) associated with their natural symbiont *Xenorhabdus* sp. TZ01, *G. mellonella* larvae died at day 1 or 2 post-infection and new IJs, named IJs_1_, emerged at day 10 ± 2. IJs_1_ were collected and washed thrice with physiological water prior to a new infection cycle started by transferring these IJs_1_ to plates containing naive (*Yersinia*-free) *G. mellonella* larvae. Ten days later, a new generation of IJs, named IJs_2_, emerged from the dead larva and so on for up to 7 consecutive infection cycles. The *Xenorhabdus* symbiont remained associated with *Steinernema* sp. MW8B throughout all infection cycles.

### Gnotoxenic EPN engineering

Axenic EPNs were obtained by manually collecting eggs from gravid *Steinernema* sp. MW8B females recovered from infected *G. mellonella* larvae prior to the term of the infectious cycle. Such axenic eggs (min 3,000) were surface sterilized with a fresh sterilization solution obtained by diluting 1ml of a 15% NaClO solution and 1ml of a 4M NaOH solution in 10ml of distilled water. The sterile axenic eggs were then suspended in YS liquid medium at 25°C during 3 days and checked for J1 larval stage development. YS medium was prepared by dissolving the following components in 1L of distilled water: 5g yeast extract (Oxoid, Basingstoke, United Kingdom); 5g NaCl (Merck, Darmstadt, Germany); 0.5g NH_4_H_2_PO_4_ (Merck); 0.5g K_2_HPO_4_ (Merck); and 0.2g MgSO_4_.7H_2_O (Merck). If no contaminants were present, IJ_1_ were deposited onto a Wouts agar plate [38] that had been freshly inoculated with 10^8^ CFU of the target bacterium in the absence of selective antibiotics. In the following days, EPNs matured to the adult stage and completed their reproductive cycle. After one week, monoxenic EPNs (IJ stage) were collected in physiological water and stored for later *Galleria* infection experiments. A similar procedure was followed to engineer polyxenic EPNs, *i.e.* EPNs harbouring two or more bacterial species.

### Microscopic observations

A 10-μl suspension of infective juveniles (IJs) was observed on a microscope slide (covered with a 18×18mm coverslip) with an epifluorescence optical microscope (Olympus BX-40-FX) with objectives 10x/0.25 (for EPNs) and 40x/0.75 (for EPNs and bacteria). Samples were observed under visible and UV light (Hg) adjusted for optimal GFP or mCherry fluorescence.

### Bacterial counts

To quantify the amount of bacteria contained in one IJs pool, and to avoid any contamination (either from the passage in *G. mellonella* or from the environment), IJs were surface sterilized following a standardized procedure. In brief, IJs were immersed in a 1.5ml eppendorf for 3 minutes with 1ml of a sterilization solution (as described previously in the M&M) with gentle agitation. After 1min centrifugation at 4000rpm in a minicentrifuge, supernatant was discarded and IJs were rinsed thrice with physiological water (0.9% NaCl). Finally, surface sterilised IJs were crushed and plated on selective agar. The number of IJs present in the pool was estimated by microscopic counting of a representative sample (50μl). Alternatively, a non-sterile method was used to assess the number of targeted bacteria associated with the IJs: *G. mellonella* cadavers were rinsed with physiological water to collect the freshly emerged IJs in suspension. The number of IJs per larva was estimated by microscopical count on 50-μl drops from this suspension. Serial dilutions of the supernatant were then plated on selective agar medium and bacteria were enumerated.

### Theoretical count of *Y. pseudotuberculosis*

In order stress out *Y. pseudotuberculosis* multiplication throughout the successive infection cycles, theoretical counts (T_Y_) that would be observed starting from the same inoculum if no bacterial division would occur have been calculated. For this, theoretical volumes of 0.5ml (V_Gm_) and 0.8nl (V_IJ_) have been assigned per *G. mellonella* larva and *Steinernema* sp MW8B IJ, respectively, and a mean EPN emergence yield of 50,000 EPNs (N_IJ_) per larva was considered. The infection dose was fixed to 125 IJs per *G. mellonella* larva. The dilution factor is the ratio V_Gm_/(V_IJ_ × N_IJ_). T_Y_ is calculated by dividing the number of *Y. pseudotuberculosis* CFUs infecting a larva by the dilution factor.

### Susceptibility testing towards *Xenorhabdus* sp. antimicrobial compounds

To estimate the resistance of enterobacteria towards *Xenorhabdus* sp. TZ01 antibiotic compound production, growth of the tested bacteria was monitored every 30 minutes during 6 hours by optical density (OD) measurement in the presence of *Xenorhabdus* culture extracts. For this purpose, LB liquid medium was inoculated with an inoculum of the target bacterium derived from a fresh culture to reach an initial OD_600_ of 0.05–0.1. Prior to inoculation, LB was supplemented with 4% or 8% (v/v) of 0.2-μm filtered supernatant of a 48h liquid culture of *X*. sp. TZ01 grown at 28°C with shaking at 150rpm (Cell Free Supernatant). *X*. sp. TZ01 liquid cultures used for supernatant preparation were stopped when OD_600_ reached 11–13. For growth curve analysis, OD_600_ was plotted into a log2 scale in order to obtain a linear graph for the exponential phase of the curve. The slope of this line (calculated with GraphPad Prism 6) defines the growth rate of a given bacterium in the defined culture conditions.

## Results

### *Yersinia pseudotuberculosis* resists to antimicrobial compounds produced by *Xenorhabdus* sp.

**Figure 1:**
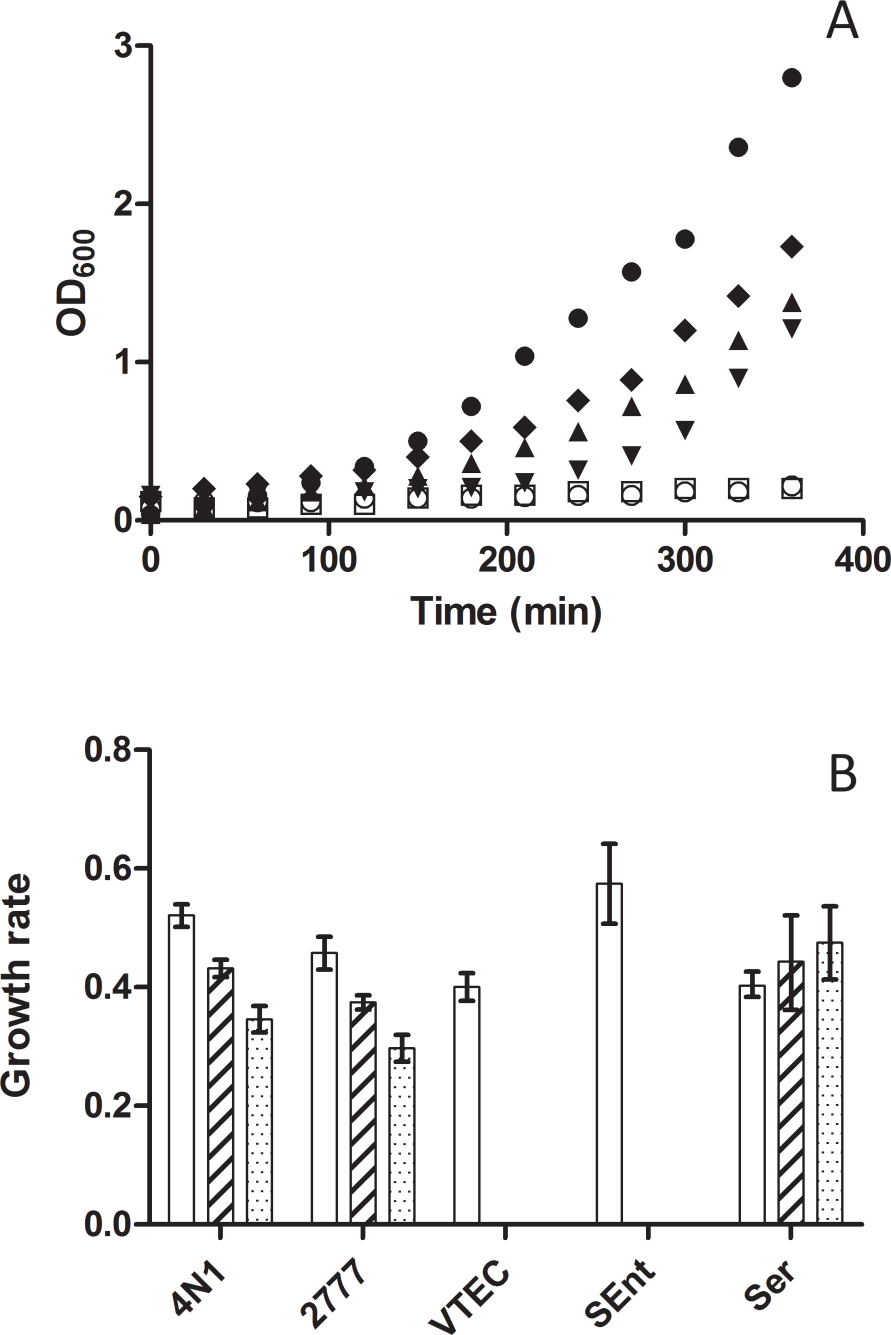
Susceptibility of various enterobacteria to antimicrobial substances produced by *X*. sp. TZ01. A: Growth curves in liquid broth of *Y. pseudotuberculosis* 4N1 (closed circles), *Y. pseudotuberculosis* 4N1 supplemented with 8% of *X*. sp. TZ01 culture supernatant (closed triangles), *Y. pseudotuberculosis* IP2777 supplemented with 8% of *X*. sp. TZ01 culture supernatant (closed diamonds), *Serratia marcescens* EE016 supplemented with 8% of *X*. sp. TZ01 culture supernatant (closed upside down triangles), *E. coli* VT01 supplemented with 8% *X*. sp. TZ01 culture supernatant (open circles) and *S*. Enteritidis SE01 supplemented with 8% of *X*. sp. TZ01 culture supernatant (opened squares). OD_600_ values were plotted every 30 minutes during 6 hours. B: Growth rates of *Y. pseudotuberculosis* 4N1 (4N1), *Y. pseudotuberculosis* IP2777 (2777), Vero-toxigenic *E. coli* VT01 (VTEC), *Salmonella* Enteritidis SE01 (SEnt) and *Serratia marcescens* EE016 (Ser) in liquid broth supplemented with either 0% (white bars), 4% (hatched bars) or 8% (dotted bars) of *X*. sp. TZ01 culture supernatant. Growth rates were calculated by plotting experimental OD_600_ values in log2 scale and taking the slope of the adjusted linear regression curve. Few or no growth was observed for Vero-toxigenic *E. coli* VT01 and *Salmonella* Enteritidis SE01 grown with either 4% or 8% of *X*. sp. TZ01 culture supernatant.

Susceptibility of several strains of *Y. pseudotuberculosis*, pathogenic *E. coli, Salmonella enterica* subsp. *enterica* serovar Enteritidis (*S*. Enteritidis) and *Serratia marcescens* towards antimicrobial compounds produced by *X*. sp. TZ01 was tested by growth curve analysis. Growth of *Y. pseudotuberculosis* (strains 4N1 and IP2777) was slightly delayed with 4% *X*. sp. TZ01 supernatant compared to a control growth without *X*. sp. TZ01 supernatant, but subsequent growth was merely unaffected when up to 8% *X*. sp. TZ01 supernatant was added. Likewise, *S. marcescens* EE016 was characterized by a delayed growth while its capacity to grow with up to 8% *X*. sp. TZ01 supernatant was unaffected. To the contrary, both *S*. Enteritidis SE01 and Vero-toxigenic *E. coli* VT01 strains were drastically inhibited with 4% *X*. sp. TZ01 supernatant (data not showed) and totally unable to grow with 8% added supernatant (Fig. 1A). Growth rates of *Y. pseudotuberculosis* 4N1 and IP2777 were both slightly affected when *X*. sp. TZ01 supernatant was added. At 4% supernatant, slopes decreased by 12% and 18% for *Y. pseudotuberculosis* 4N1 and IP2777, respectively. These values dropped respectively by 30% and 35% when 8% of *X*. sp. TZ01 supernatant was added. *S. marcescens* EE016 growth was unaffected with either 4% or 8% added *X*. sp. TZ01 supernatant (Fig. 1B).

### *Yersinia pseudotuberculosis* colonizes the gastro-intestinal tract of EPNs and survives long-term EPN storage

To assess the ability of *Y. pseudotuberculosis* to colonize *Steinernema* sp. MW8B, 7 independent *Galleria mellonella* infection experiments were conducted with a *Y. pseudotuberculosis* GFPmut2-labelled strain (4N1G strain) (Table 2).

**Table 2:** Summary of *G. mellonella* infection experiments.

**Infection Cycle**	**IJ emergence**	**IJ fluorescence**	**IJ infectivity**	**IJs/larva**	***Yp* CFU/IJ**	**Total *Yp* count**
1	7/7	7/7	5/7	50000 +/− 7500	5,0 × 10^3^ +/− 0,8 × 10^3^	2,5 × 10^8^ +/− 0,3 × 10^8^
2	5/7	3/7	4/7	ND	ND	ND
3	4/7	2/7	3/7	ND	ND	ND
4	3/7	2/7	3/7	40800 +/− 6366	8,6 × 10^3^ +/− 1,6 × 10^3^	3,5 × 10^8^ +/− 0,5 × 10^8^
5	3/7	1/7	2/7	ND	ND	ND
6	2/7	1/7	1/7	ND	ND	ND
7	1/7	1/7	NA	1022 +/− 247	5,6 × 10^3^ +/− 1,8 × 10^3^	5,7 × 10^6^ +/− 1,4 × 10^6^

Seven *G. mellonella* larvae were injected with 10^6^ CFU of the GFP-labelled *Y. pseudotuberculosis* strain 4N1G (*Yp*) and incubated with ca. 125 *Steinernema* sp. MW8B nematodes (Infective Juvenile stage, IJ) associated with their natural *Xenorhabdus* sp. TZ01 symbiont as described in M&M. After completion of the first infectious cycle, *Steinernema* sp. MW8B progeny emerging from one death larva was collected, characterized according to several parameters and used to infect naïve (*Yp*-free) *G. mellonella* larvae thereby initiating a new infection cycle, and so on for 7 successive cycles. Column 2–4, number of experiments in which emerged IJs fitted the property featured on the top row; column 5, mean number of IJs emerged from one dead larva; column 6, mean count of *Yp* CFU retrieved from one crushed IJ; column 7, total count of *Steinernema* sp. MW8B-associated *Yp* CFU generated from one *G. mellonella* larva after successful cycle completion. ND, not determined; NA, not available. The *Xenorhabdus* sp. TZ01 symbiont was still present after each successful cycle completion.

**Figure 2:**
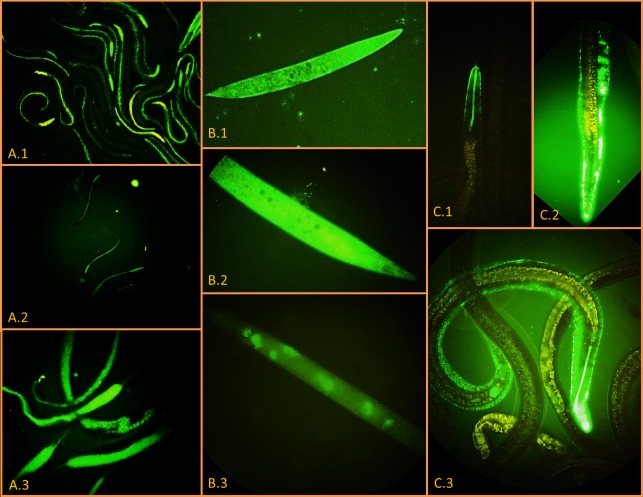
Epifluorescence microscope pictures of GFP-labelled *Y. pseudotuberculosis* 4N1G in *Steinernema* sp; MW8B EPNs. A. EPNs emerging from dead moth larvae after 1 (A.1), 4 (A.2) and 7 (A.3) consecutive infection cycles (100× magnification). B. IJs collected after the first infection cycle and stored at 4°C in physiological water for either 8 (B.1, B.2) or 42 days (B.3) (400× magnification). C. IJs collected after the first infection cycle and stored at 28°C in physiological water for 98 days. C.1, enlarged view of the mouth; C.2, enlarged view of the anus; C.3, whole IJ body (400× magnification, 800× magnification for enlarged view).

In all experiments, *Steinernema* sp. MW8B IJs_1_ exhibited GFPmut2 fluorescence along the entire length of their gut (Fig. 2A.1). However, in 2 out of 7 experiments (29%), IJs1 emerged from *Y. pseudotuberculosis* 4N1G-infected *G. mellonella* larvae failed to invade and kill new naive *G. mellonella* larvae in spite of the fact that they were massively colonized by *Y. pseudotuberculosis* 4N1G as attested by the bright GFP fluorescence they displayed. In 2 out of 7 experiments (29%), IJs that were both fluorescent and infective emerged from dead *G. mellonella* cadavers after 4 consecutive infection cycles (Fig. 2A.2). One experiment (14%) led to the emergence of fluorescent/infective IJs after the fifth infection cycle and were so even after 7 consecutive infection cycles which lasted 14 weeks (Fig. 2A.3).

Directly after the first emergence, freshly emerged IJs_1_ were stored at 4°C, 16°C and 28°C in physiological water. IJs_1_ were observed in epifluorescence microscopy to monitor the presence of *Y. pseudotuberculosis* 4N1G. These observations were made every day during the first week post emergence (PE) then once a week during 13 weeks. At 4°C, stored IJs_1_ did not survive a week and were all dead by day 8 PE (Fig. 2B.1 and 2B.2). Nevertheless, *Y. pseudotuberculosis* 4N1G was still alive—and did even multiply slowly inside the IJs cadavers—since GFP fluorescence was still observed 6 weeks PE (Fig. 2B.3). No differences were observed between IJs_1_ stored either at 16°C or at 28°C. In these samples, microscopic observations showed that *Y. pseudotuberculosis* 4N1G was still present inside IJs_1_ of *Steinernema* sp. MW8B, either in the gut or in the inter-cuticular space, after 14 weeks of storage at either 16°C or 28°C (Fig. 2C).

To check the ability of *Y. pseudotuberculosis* 4N1G to remain associated with *Steinernema* sp. MW8B after several infection cycles, IJs_4_ were also kept at 28°C. At 3 weeks PE, 23 +/− 3 IJs_4_ were crushed and counted on selective agar plates. An average of 5.0 × 10^3^ CFUs of *Y. pseudotuberculosis* 4N1G per IJs_4_ was measured. Compared to IJs_4_ at 0 day PE (8.6 × 10^3^ CFU/IJs_4_), the number of *Y. pseudotuberculosis* 4N1G CFUs per IJ decreased by 41%. However, quantitative results obtained for IJs_4_ at week 3 PE and IJs_1_ at day 0 PE are comparable.

**Figure 3:**
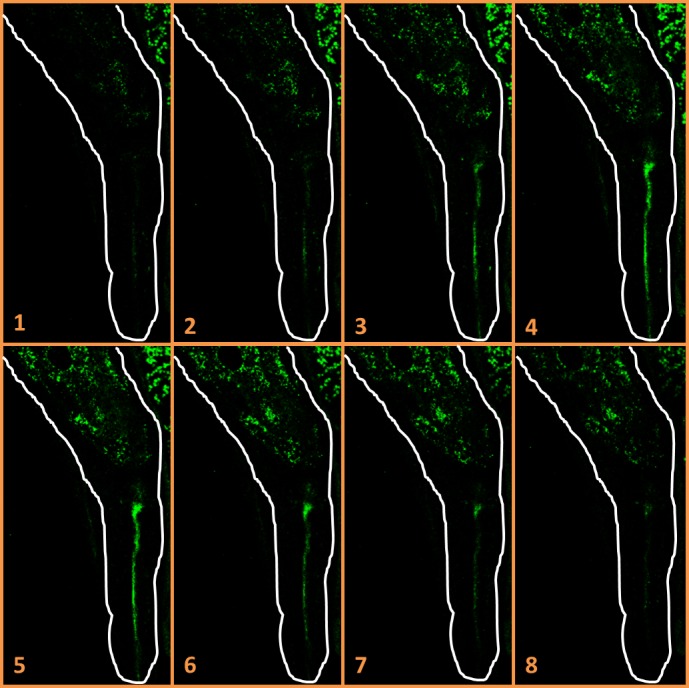
Localization of *Y*. *enterocolitica* YE03 in *Steinernema* sp. MW8B EPNs emerged from an infected larva. Confocal microscope slides in Z-axis (numbered from 1 to 8) of a *Steinernema* sp. MW8B EPN colonized by *Y. enterocolitica* YE03 emerged from the second infection cycle. GFP-labeled bacteria localize in the mouth and in the gut lumen. EPN borders are drawn in white (800× magnification).

Similar *G. mellonella* infection experiments were conducted three times with a GFPmut2-labelled *Y. enterocolitica* O:3 strain (YE03). This strain is more sensitive towards antimicrobials produced by *X*. sp. since *Y. enterocolitica* O:3’s growth is totally inhibited with the presence of 8% of *X*. sp. supernatant (data not shown). Microscopic observations showed EPN colonization by *Y. enterocolitica* YE03 during at least 2 consecutive infection cycles for one out of the three experiments conducted. Confocal microscopic observations localized *Y. enterocolitica* YE03 in the gut lumen after 2 infection cycles (Fig. 3). However, no GFPmut2 fluorescence was observed after 3 consecutive infection cycles with *Y. enterocolitica* YE03. GFPmut2-labelled *Escherichia coli* VT03 (vero-toxigenic O157 strains), GFPmut2-labelled *Salmonella* Enteritidis SE03 and an unlabelled tetracycline resistant *S. marcescens* EE016 strain were subjected to similar *G. mellonella* infection cycle experiments. None of these 4 *Enterobacteriaceae* demonstrated *Steinernema* sp. MW8B colonisation capacity. This was evidenced by the lack of GFPmut2 fluorescence in IJs_1_ in the *E. coli* VT03 and *S*. Enteritidis SE03 experiments. No single IJ_1_ emerged from *G. mellonella* larvae injected with *S. marcescens* EE016.

### EPNs support dramatic multiplication and dissemination of *Yersinia pseudotuberculosis*

**Figure 4:**
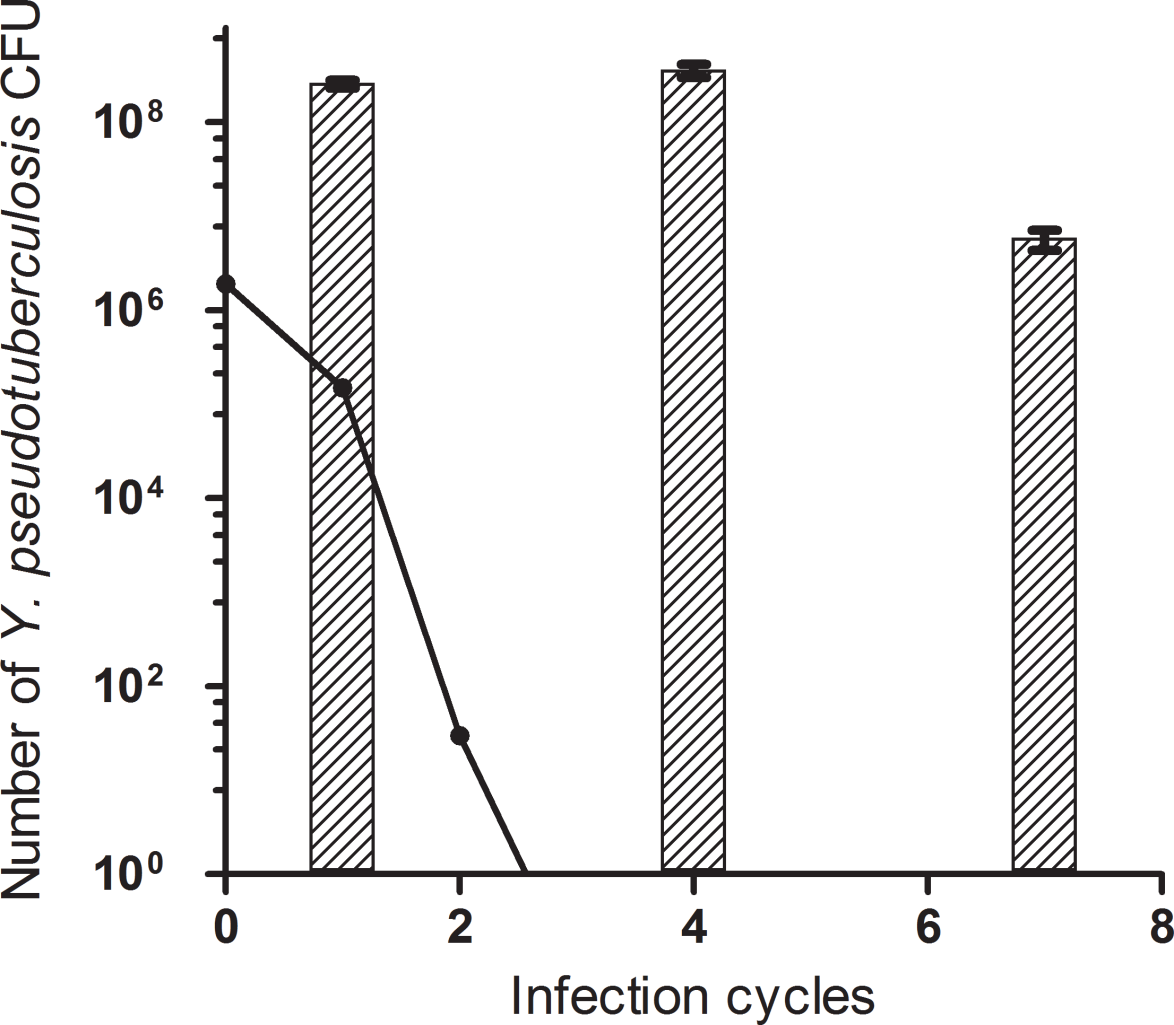
Growth of *Y. pseudotuberculosis* 4N1G during EPN’s infection cycles. The hatched bars show the total counts of *Y. pseudotuberculosis* 4N1G CFUs retrieved from IJs emerged from a dead moth larva after 1, 4 and 7 consecutive infection cycles (data from table 2). The straight line shows the theoretical counts that would be observed starting from the same inoculum if no bacterial division would occur. For this calculation, theoretical volumes of 0.5ml and 0.8nl have been assigned per *G. mellonella* larva and *Steinernema* sp MW8B IJ, respectively, and a mean EPN emergence yield of 50,000 EPNs per larva has been considered (see M&M).

To confirm quantitatively the maintenance of *Y. pseudotuberculosis* 4N1G in the experimental model, CFU counts were determined at different time points (Table 2). After the first infection cycle, an average of 5.0 × 10^3^ *Y. pseudotuberculosis* 4N1G CFUs per *Steinernema* sp. MW8B IJ were found. Similar counts were determined during 7 consecutive infection cycles, with an average of 8.6 × 10^3^ CFUs of *Y. pseudotuberculosis* 4N1G per IJ still found after the 4^th^ infection cycle and 5.6 × 10^3^ CFUs after the 7^th^ infection cycle (Table 2). Knowing the number of CFU per IJ and the number of IJs emerged from dead larvae, we calculated the total increase in *Y. pseudotuberculosis* 4N1G counts after the various infection cycles. Starting with 1.9 × 10^6^ CFUs of *Y. pseudotuberculosis* 4N1G directly injected in the larva, *Y. pseudotuberculosis* 4N1G counts after one cycle increased by two orders of magnitude and reached 2.5 × 10^8^ CFUs. These counts were similar after the fourth infection cycle (3.5 × 10^8^ CFU) and started to decrease from the 7^th^ infection cycle (5.67 × 10^6^ CFU). This confirms our model predictions which suggest that in the absence of active multiplication, *Y. pseudotuberculosis* 4N1G counts would drastically decrease and would become undetectable after two infection cycles (Fig. 4). The experimental counts hence reflect an active multiplication of *Y. pseudotuberculosis* 4N1G in the studied laboratory model.

### *Yersinia pseudotuberculosis* cannot replace *Xenorhabdus* sp. TZ01 as EPN symbiont

**Figure 5:**
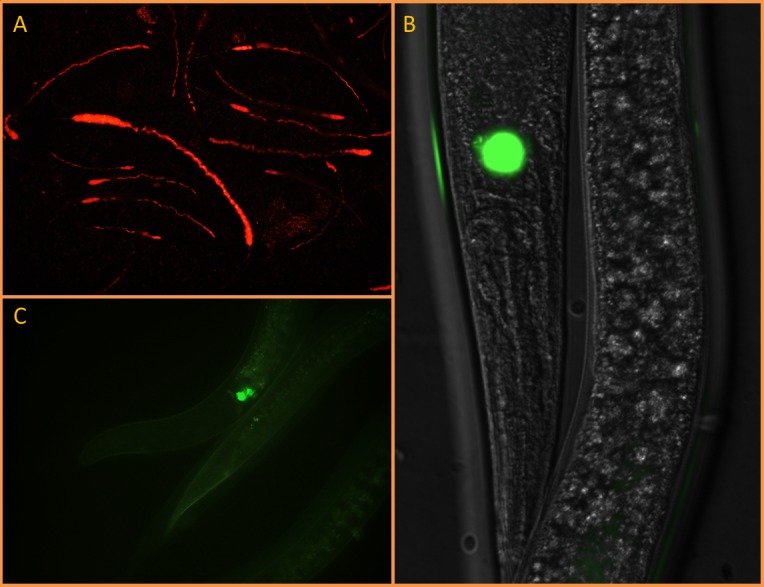
Differential localization of *Y. pseudotuberculosis* 4N1C and *X*. sp. TZ03 in *Steinernema* sp. MW8B nematodes. Epifluorescence microscope pictures showing axenic EPNs artificially fed on (A) plate-grown red fluorescent *Y. pseudotuberculosis* 4N1C localizing in the gut (100× magnification) or (B) plate-grown green fluorescent *X*. sp. TZ03 localizing in a symbiotic vesicle (400× magnification). The latter was still localized in the symbiotic vesicle after 2 consecutive infection cycles on *G. mellonella* larvae (C) (800× magnification)

After having demonstrated the colonisation and multiplication capacity of *Y. pseudotuberculosis* 4N1G in the gut of *Steinernema* sp. MW8B IJs, we wondered whether *Y. pseudotuberculosis* 4N1G could substitute for *X*. sp. TZ01 as a bacterial symbiont in this EPN species. To address this question, we obtained axenic *Steinernema* sp. MW8B EPNs by collecting surface sterilised eggs from gravid females. Prior to *G. mellonella* infection, axenic EPNs were incubated with the mCherry-labelled *Y. pseudotuberculosis* 4N1C strain on Wouts Agar plates in order to obtain IJs exclusively colonised by *Y. pseudotuberculosis* 4N1C. A pool of such “monoxenic” IJs displaying red fluorescence (Fig. 5A) was divided into 4 equal groups. Two groups were incubated separately with 6 *G. mellonella* larvae in empty containers. One group was deposited onto a sterile Wouts Agar plate with 4 *G. mellonella* larvae and the last group was deposited onto a Wouts Agar plate without any larva.

At day 3 post infection (PI), only *G. mellonella* larvae grown on Wouts Agar were found dead. At day 8 PI, all *G. mellonella* were dead. Three *G. mellonella* larvae recovered from Wouts agar plates showed emergence of EPNs. The emerged EPNs displayed no mCherry fluorescence when observed microscopically. When crushed and plated onto selective agar plates, no *Y. pseudotuberculosis* 4N1C could be retrieved from these EPNs neither. At day 10 PI, emergence of EPNs could be observed in 2 larvae incubated in empty containers, but again none of the emerged EPNs exhibited red fluorescence and no *Y. pseudotuberculosis* 4N1C could be retrieved after EPN crushing and plating on selective agar. In contrast, EPNs grown freely on Wouts agar still exhibited red mCherry fluorescence 10 days after plating. The same experiment was conducted with the GFPmut2-labelled *X*. sp. TZ03. Microscopic observations showed not only that *X*. sp. TZ03 was able to colonise the symbiotic vesicle of *Steinernema* sp. MW8B axenic EPNs (Fig. 5B), but also that *X*. sp. TZ03 maintained in its host after 2 consecutive cycles (Fig. 5C) and probably much more (not tested).

## Discussion, Conclusion, and Perspectives

Compared to other enterobacteriaceae tested so far, the capacity of *Y. pseudotuberculosis* 4N1 to colonize *Steinernema* sp. MW8B is remarkably efficient and suggests that a number of biological functions required for its successful dissemination through this host during and between infection cycles are present and functional in this bacterium. We showed that enterobacteria sensitive to the antibiotics secreted by *Xenorhabdus*. sp. TZ01 have no ability to colonize the EPN gut. Two strains of *Y. pseudotuberculosis* (4N1 and IP2777) as well as one *S. marcescens* strain (EE016) isolated from a *Steinernema* sp. MW8B-infected *G. mellonella* larva were found naturally resistant to *X*. sp. TZ01 secreted antibiotics and were tested for their ability to colonize *Steinernema* sp. MW8B EPNs with the model developed herewith. The growth rate of both *Y. pseudotuberculosis* strains was slightly affected by the presence of *X*. sp. TZ01 supernatant, while the *S. marcescens* EE016 was not. Likewise, *Ochrobactrum tritici* strain EE10.1 isolated from a *Steinernema* sp. MW8B-infected *G. mellonella* larva in our laboratory displayed a similar capacity to resist to *X*. sp. TZ01 antibiotics (data not shown). Despite this capacity, *S. marcescens* EE016 was unable to sustain EPNs life cycle completion since no IJ emergence occurred from *S. marcescens*-injected *G. mellonella* larvae. This suggests that *Serratia* and *Ochrobactrum* may accidentally reach the gut of *Steinernema* sp. MW8B but are unlikely able to colonize and multiply within the EPN gut as *Yersinia pseudotuberculosis* does. It has been shown that *Serratia marcescens* uses the type VI secretion system to neutralize bacterial competitors [39]. Injection of 10^6^ *S. marcescens* CFUs may therefore impair *Xenorhabdus* growth in *G. mellonella* larvae. This could explain why *Steinernema* sp. MW8B cannot complete its reproductive cycle within *S. marcescens*-infected *G. mellonella. S. marcescens* has been shown to be pathogenic towards the free-living nermatode *Caenorhabditis elegans* but beneficial to the entomopathogenic nematode *C. briggsae* [40,41]. Zhang et al. reported the isolation of a new *Serratia* species (*S. nematodiphila*) from the EPN species *Heterorhabditidoides chongmingensis* and proposed that *S. nematodiphila* may have evolved to a symbiotic species, possibly after horizontal gene transfer [42,43]. Dixenic associations have been described, such as *P. luminescens* and *Ochrobactrum spp.* found together in tropical species of *Heterorhabditis* [44]. Genomic comparison between *S. nematodiphila* and other (non-symbiotic) *Serratia spp*. could provide interesting insights in the discovery of genes involved in the symbiotic association with EPNs.

In this study we showed that *Y. pseudotuberculosis* 4N1G is able to colonize and maintain for several generations inside a *Steinernema* species for long-term periods (14 weeks). Quantitative data showed that EPNs support efficient multiplication of *Y. pseudotuberculosis* 4N1G during this period. Indeed, counts of *Y. pseudotuberculosis* CFUs carried away by EPNs emerged from dead larvae are roughly multiplied by a factor 10^3^ at the term of each infection cycle, a number which is probably underestimated as it does not take into account *Y. pseudotuberculosis* bacteria left over in the dead cadaver. *Y. pseudotuberculosis* 4N1G colonizes mainly the gut of *Steinernema* sp. MW8B but can be found in the inter-cuticular space as well after 3-month storage in physiological water. The localisation of *Y. pseudotuberculosis* 4N1G in *Steinernema* sp. MW8B IJs’ gut is quite different from the normal localisation of the symbiotic *Xenorhabdus* sp. TZ03. Indeed, the natural niche of *Xenorhabdus* inside its *Steinernema* host–before infecting an insect prey–is a so-called symbiotic vesicle located along and separated from the EPN gut [3]. Our observations on EPN colonization are consistent with axenic EPNs experiments, which demonstrated that *Y. pseudotuberculosis* 4N1G does not replace the *X*. sp. TZ01 symbiont during EPN infection cycle but more likely hijack the symbiotic relationship between *Xenorhabdus* and EPNs. Indeed, when the natural symbiont of *Steinernema* sp. MW8B is absent, EPNs colonized by *Y. pseudotuberculosis* 4N1C alone are unable to develop properly in a *G. mellonella* larva. In control experiments where axenic EPNs are supplemented with GFP-labelled *X*. sp. TZ03, EPNs recover their ability to indefinitely multiply and feed on *G. mellonella* larvae. Moreover, unpublished results showed that IJs colonized by both *X*. sp. and *Y. pseudotuberculosis* strains labelled with two different fluorescent protein markers do contain both bacteria.

The mini-Tn*5* transposon used to tag *Y. pseudotuberculosis* 4N1G and 4N1C in our experiments was mapped in the fimbrial A protein gene. This gene is found in two intact copies in the *Y. pseudotuberculosis* genome meaning that the protein is probably still expressed in the GFP-tagged strains. Fimbrial proteins are known to act as colonization factors for *Y. pseudotuberculosis* and are involved in the attachment to epithelial cells [45]. The capacity of the 4N1G and 4N1C tagged strains to colonise the EPN’s gut, as demonstrated throughout our study, argue in favour of a non-detrimental effect of the transposon insertion compared to the wild-type strain.

Interestingly, it has been shown that *Y. pseudotuberculosis* and *Y. pestis* – the etiological agent of plague – can infect or form a biofilm mainly around the head of *Caenorhabditis elegans*, a well-studied nematode laboratory model [46,47,48]. Given the fact that *Y. pestis* evolved quite recently from *Y. pseudotuberculosis* [49], it would be interesting to know whether *Y. pestis* can also resist to antimicrobial substances produced by *Xenorhabdus*/*Photorhabdus* spp. and colonize EPNs. If these findings turn to have an environmental significance, it would provide new insights in the understanding of long-term persistence of *Y. pestis* in plague endemic areas worldwide [28,50].

Future work should focus on the identification of *Yersinia* genetic determinants required to colonize and maintain inside EPNs. Several genes shared by *Yersinia* and the EPN’s natural symbionts are good candidates to play this role such as the phospholipase A encoded by *yplA*, structural genes of the type 6 secretion system (T6SS) and possibly others [10]. Likewise, genome comparisons between *Serratia, Ochrobactrum, Yersinia* and *Xenorhabdus* should help deciphering the critical genetic determinants required for EPN colonization.
